# Safe internalization of external cholecystostomy drain by endoscopic ultrasound-guided drainage using a lumen apposing metal stent with endoscopic clipping

**DOI:** 10.1055/a-2725-7066

**Published:** 2025-11-10

**Authors:** Saburo Matsubara, Risa Sunada, Tomohiro Arai, Keito Nakagawa, Noriko Murakami, Morito Ikeda, Naosuke Kuraoka

**Affiliations:** 1 Department of Gastroenterology and Hepatology, Saitama Medical Center, Saitama Medical University, Saitama, Japan

An 83-year-old male with metastatic pancreatic cancer derived from an intraductal papillary mucinous neoplasm undergoing chemotherapy was admitted for severe acute calculous cholecystitis with septic shock and acute renal failure. Gangrenous cholecystitis was suspected because a fluid collection near the gallbladder fundus with wall disruption was detected by computed tomography and ultrasound. Following initiation of hemodialysis and administration of broad-spectrum antibiotics and vasopressor agents, urgent percutaneous cholecystostomy (PC) was performed with an 8.5-Fr pigtail tube. After symptom resolution, the patient requested removal of the external drain prior to discharge. However, simple removal carried a risk of bile leakage because the fistula might not mature owing to poor nutritional status and mild ascites. Therefore, internalization by endoscopic ultrasound-guided gallbladder drainage (EUS-GBD) using a lumen apposing metal stent (LAMS) was attempted two weeks after admission ( Video 1 ). A 10-mm × 10-mm electrocautery-enhanced LAMS (Hot AXIOS; Boston Scientific, Marlborough, Massachusetts, USA) was deployed from the gastric antrum under EUS and fluoroscopy guidance with luminal dilation achieved by continuous saline injection through the external tube ( Fig. 1 ). A slim therapeutic endoscope (EG-840TP; Fujifilm, Tokyo, Japan) with a 3.2-mm working channel was then inserted into the gallbladder via the AXIOS after balloon dilation. Subsequently, the external tube was removed, followed by immediate closure of the puncture site with endoscopic clips (SureClip; Micro-Tech, Ann Arbor, Michigan, USA) ( Fig. 2 ). The post-procedural course was uneventful.

Safe internalization of external cholecystostomy drain by EUS-guided drainage using a lumen apposing metal stent with endoscopic clipping.Video 1

**Fig. 1:**
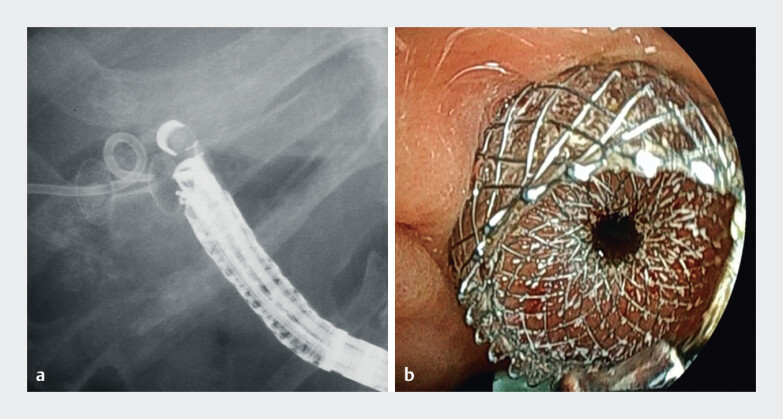
Endoscopic ultrasound-guided gallbladder drainage using a 10-mm × 10-mm Hot AXIOS. **a** Fluoroscopic image. **b** Endoscopic image.

**Fig. 2:**
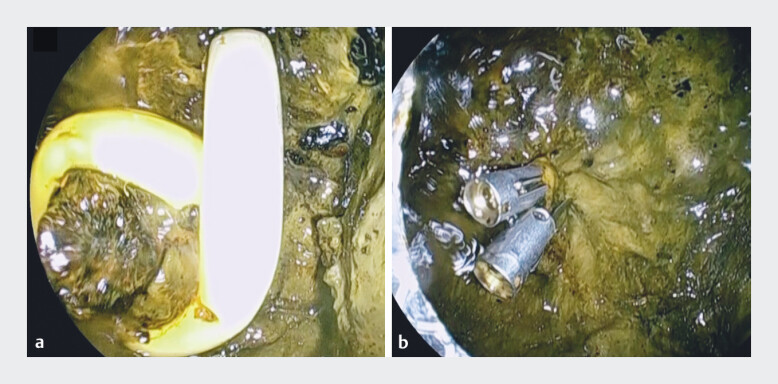
Endoscopic closure of the puncture site using endoscopic clips. **a** The pigtail tip of the external tube is visible. **b** Closure of the puncture site with clips immediately after tube removal.

Although EUS-GBD for acute-cholecystitis unsuitable for surgery is the gold standard nowadays, patients with gangrenous cholecystitis or unstable vital signs are contraindicated for safety reasons 1 . Conversion from PC to EUS-GBD is standard care for these patients 2 ; however, bile leakage is of concern in cases with suspected immature fistula 3 . EUS-GBD using a LAMS with clipping can be a useful option for safely removing the external drain in such patients.

Endoscopy_UCTN_Code_TTT_1AS_2AH

## Notes

Endoscopy E-Videos https://eref.thieme.de/e-videos
*E-Videos* is an open access online section of the journal *Endoscopy* , reporting on interesting cases and new techniques in gastroenterological endoscopy. All papers include a high-quality video and are published with a Creative Commons CC-BY license. Endoscopy E-Videos qualify for HINARI discounts and waivers and eligibility is automatically checked during the submission process. We grant 100% waivers to articles whose corresponding authors are based in Group A countries and 50% waivers to those who are based in Group B countries as classified by Research4Life (see: https://www.research4life.org/access/eligibility/ ). This section has its own submission website at https://mc.manuscriptcentral.com/e-videos .
